# Supplementary material for: Left powerless: A qualitative social media content analysis of the Dutch #breakthesilence campaign on negative and traumatic experiences of labour and birth
Source: PLoS One. 2020 May 12;15(5):e0233114. doi: 10.1371/journal.pone.0233114 (PMC7217465; doi:10.1371/journal.pone.0233114)
Supplement: Attachment — Submitted filename: Response to Reviewers.pdf [file pone.0233114.s001.pdf]

16 March 2020

Manuscript Number: PONE-D-19-33121

Dear Mr Fischer,

We thank you and the reviewers for their constructive comments on our manuscript entitled *Left powerless: a social media content analysis of the Dutch #breakthesilence campaign on negative and traumatic experiences of labour and birth* which we submitted for publication in PLOS ONE and for giving us the opportunity to revise the manuscript in light of these comments.

Please find a detailed response to the comments below. We copied the comments of the reviewers and wrote our response in *Italic* next to each comment. We hope you are in agreement with our edits to the original manuscript and look forward to your reply regarding publication.

Yours sincerely, on behalf of the authors,

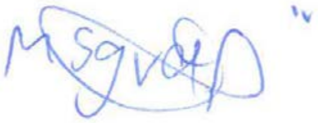

Marit van der Pijl, MSc

E: m.vanderpijl@amsterdamumc.nl (MP)

T: +31650892910

Detailed response to the editor and the reviewers

**Editor**	
Comment	*Response*
Please ensure that your manuscript meets PLOS ONE's style requirements, including those for file naming.	*We thank the reviewer for addressing the requirements for submitting the revision.*<br><br>*All style requirements are checked and where necessary adjusted according to the PLOS ONE style templates.*
In your data availability statement, you write, "All #genoeggezwegen files analysed for the study are available and can be found at the Facebook page of Birth Movement NL (https://www.facebook.com/pg/GeboorteBeweging/photos/?tab=album&album_id=1149487385089171&ref=page_internal)." We note that the content on this Facebook page is at least partially restricted. Please deposit your underlying data to a dedicated data repository. A list of potential repositories, including ones with mechanisms for data access control is available here: https://journals.plos.org/plosone/s/data-availability#loc-recommended-repositories. Alternatively, if the data cannot be deposited, please discuss (1) the reasons for the restrictions and (2) a data-sharing mechanism such that qualified researchers could request the data. Note that it is not sufficient for a single author to be the sole individual responsible for ensuring data access.	*We double checked the link to the Facebook page of the Birth Movement. The link seems to be correct. However, it takes some time to load all files due to the large number of pictures on the website. When scrolling down, new files become visible. It is possible that this caused the restrictive view.*<br>*We are willing to compute and upload a PDF file including all transcriptions (only text files), if required by the editor.*
We note that one or more authors are affiliated with Hechte Band or Stichting Geboortebeweging. In your competing interests statement, please discuss the relationship with this company and organization to the topic of the study. Note that PLOS ONE's competing interests' policy requires authors to discuss anything that could be perceived as a competing interest. For more information on what to declare, see https://journals.plos.org/plosone/s/competing-interests#loc-what-to-declare.	*Client involvement in research is becoming more and more important. We believe that, given the topic of our research, the involvement of client representatives was essential to ensure the quality of our study. We adjusted the competing interest statement in the manuscript as follows:*<br><br>*We declare that the organizations Hechte Band and Geboortebeweging did not provide funding to carry out this study. Two authors (TL and RV) affiliated with these organizations were part of the research team representing the client. TL is a member of Geboortebeweging, a clinical psychologist and a PhD student (GGzE & Vrije Universiteit). RV is also a member of Geboortebeweging and is a certified babywearing consultant and bonding coach (Hechte Band). Both authors were involved in managing the #GG campaign in 2016, in which they also participated themselves.TL and RV were involved in the research process from the start. They provided feedback on the research proposal and design of the study and contributed to discussing the findings within the research team and writing the paper, all from a client point of view. They were not involved in data collection or analysing the data.*

**Reviewer #1**	
Comment	*Response*
Lines 86 and 585 note that women were asked to write their experiences "on a piece of paper". If this was the process, who collected and posted responses online? It may be that they were asked to post their experiences (along with the hashtag) on social media? If not, who asked them to write it on the paper? Where? Who was asked? Also, it's at this juncture that I'm wondering what platform(s) was/were suggested (i.e. facebook, twitter, instagram, etc.). Similarly, at line 123 it is noted that stories were collected from FB but also via email? This further confuses where/how stories were collected by the movement. The campaign process requires some clarity in order to inform data collection.	*First of all, we would like to thank the reviewer for the suggestions to improve our manuscript.*<br><br>*We recognize that the campaign process was not clearly described in the manuscript and apologize for this. We clarified the campaign process in the Introduction section,*<br>*see page 4 line 86 to 94 in the unmarked version of the manuscript. In the Methods section we made adjustments,*<br>*see page 6 line 128 to 136. Also, we made adjustments in the Discussion, see page 27-28 line 579 to 580 and 586 to 587.*
Line 136 - I'm assuming that identifiable information in the women's stories as well as social media platform id's were also removed/deidentified? Also, at this point in the manuscript I'm a bit confused as to whether you're assessing images and text or just text.	*As only the pictures shared through email with the Birth Movement were included in the study, information on social media platform id's is not applicable. This is clarified in the description of the campaign process, see above. Only textual content was assessed for this research.*<br><br>*We specified the steps we took to guarantee the privacy of women in our study in the section on ethical considerations, see page 6 line 139 to 145.*
Beginning at line 466, the authors bring in another study regarding higher income settings - This is not well integrated and it seems out of place in the discussion.	*We agree with the reviewer that this study is not well integrated in the Discussion. Therefore, we decided to remove this section from the Discussion.*
"Women reported a lack of informed consent, not being taken seriously and not being listened to, lack of compassion and use of force; this left them feeling powerless. This indicates that disrespect and abuse during labour and birth do happen in the Netherlands, although the current study gives no insight into prevalence" - This study doesn't confirm disrespect and abuse, it only confirms that the women perceived their experiences as such. One of the limitations, as noted, as that we don't know the circumstances/timing/etc. of the experiences. Women's perceptions of their experiences are no less valuable but we have to be careful what we claim from the findings. This is prevalent throughout. The paper can be framed to describe women's perceptions of care rather than proof of abuse.	*We understand the concerns of the reviewer and we fully agree that the paper should represent women's experience only, as there is no further information from other perspectives available. Therefore, the aim of the study was focused on understanding the experiences of women participating in the #GG campaign, rather than understanding occurrences of disrespect and abuse. Also, in the data analysis we addressed the way in which we would formulate the findings section, showing that we solely represent women's words. Furthermore, as the reviewer also mentioned, we described the limited perspective as one of our limitations.*<br>*To further emphasize the perspective of women, we adjusted the sentence mentioned by the reviewer to: ''This may indicate that disrespect and abuse during labour and birth do happen in the Netherlands, although the current study gives no insight into prevalence.''*<br><br>*We also adjusted sections throughout the paper. Some examples: page 5 line 117 to 119, page 8 line 174, page 13 line 246 to 247, page 28-29 line 605 to 607.*

**Reviewer #2:**	
Comment	*Response*
This is an interesting and informative piece of scholarship that uses social media content to contribute to understanding of traumatic labor and birth experiences. In many respects, the manuscript is outside my area of expertise, so I am unable to comment on some of the substance. My main qualification to review this manuscript is in terms of methods. Therefore, I am going to focus my review on that component of the research. While I do see some issues that are important and must be addressed, I ultimately think they are issues that can be sorted out.	*We thank the reviewer for the comments and suggestions to improve our manuscript.*
I am concerned that the term "content analysis" is **misused** in the methods section. The term "content analysis" is typically reserved for quantitative approaches, as is described in the following works: .<br>- Krippendorff, K. H. (2013). Content Analysis: An Introduction to Its Methodology (Third Edition). Sage Publications, Inc.<br>- Neuendorf, K. A. (2017). The content analysis guidebook (Second edition). SAGE.<br>- Riffe, D., Lacy, S., & Fico, F. G. (2005). Analyzing Media Messages: Using Quantitative Content Analysis in Research (2nd ed.). Lawrence Erlbaum Associates.<br>The qualitative process described here should be referred to as "textual analysis" or "qualitative content analysis" or by some other term to avoid confusion. I will note that it is referred to in these kinds of terms in other places in the manuscript, so this should frankly be an easy fix.	*We thank the reviewer for the literature suggestions on content analysis that were very helpful for learning more about approaches to content analysis.*<br><br>*As suggested by the reviewer, we changed the term ''content analysis'' to "qualitative content analysis" throughout the paper, to clarify that we only used a qualitative approach in our study.*
This brings me to my second – and perhaps more substantive – critique of the work. While it is billed as a qualitative analysis, extensive quantitative results are reported. It is generally considered to be inappropriate to report figures from a qualitative textual analysis as though a quantitative process was conducted, because quantitative content analysis has certain requirements to ensure reliability and validity.	*We are grateful for the reviewer alerting us to unclarity about the respective roles of qualitative and quantitative analysis in this study. We want to emphasize that we did not perform a quantitative analysis; only a qualitative analysis was used to analyse the stories.*<br>*The 'quantitative' section on the types of caregivers and interventions was meant to provide additional information about the characteristics of the stories, as no information is available on the characteristics of the participants themselves. The typology of Bohren et al. allowed us to use predetermined codes based on existing theory to gain insight in what types of mistreatment were described in the stories.*<br>*We realize that terms directed and conventional content analysis were not correctly used. Therefore, we adjusted the description of our approach throughout the manuscript. To summarize:*<br>- *In the data analysis, we specified two qualitative methods: (1) a deductive coding procedure (coding based on typology of Bohren et al.) and (2) inductive coding procedure (open coding). See page 7-8 line 148 to 173. The Results are structured accordingly.*<br>- *The types of caregivers and interventions that we identified in the stories are mentioned shortly at the*

	beginning of the Results section, see page 11, line 212 to 217.
The use of MAXQDA Analytics Pro software is mentioned, but it is unclear what that software was used for. Was it used to automate the analysis process, or was it simply the platform on which coding was conducted? If human-coded quantitative content analysis was conducted, then intercoder reliability scores MUST be reported. If an algorithmic content analysis was conducted, it must be described in more detail.	*MAX-QDA is used to manually assign codes and categories. To clarify this, we adjusted the beginning of the data analysis section, see page 7 line 148 to 150.*<br><br>*The first 50 stories were coded and categorized by two researchers together (MP and MH). Any discrepancies in coding were discussed and consensus was found. Thereafter the remaining stories were analysed by only one researcher (MP). Therefore, an intercoder reliability score is not necessary. We clarified this in the manuscript, see page 7 line 165 to 167.*
I am unclear what this passage means on p. 7 (Line 155): "… a conventional content analysis, in which categories were derived from the data…" Generally speaking, in a conventional quantitative content analysis, coding categories are determined before data are collected. The authors may wish to briefly (one paragraph or so) reflect on the strengths and weaknesses of inductive vs. deductive processes in textual analysis.	*As we adjusted the description of our approach, the term conventional content analysis is no longer used.*<br><br>*We used both a deductive as an inductive approach in the qualitative data analysis. We explained this in the data analysis section, see page 7 line 148 to 173. Furthermore, we discussed our approach in the strengths and limitations section in the Discussion, see page 27 line 573 to 578.*
The authors write: "Five major themes emerged from the stories." How were these this determined? There needs to be more transparency about how these themes were decided upon.	*The themes were addressed in the data analysis section as 'main categories'. We apologize for this confusion. We adjusted the term in the data analysis section, see page 7 line 169 to 170.*
While it is backed up by a citation, the statement "Currently, little use is made of social media content as data for health care research, despite its great potential due to the huge volume of health care experiences shared online" seems implausible. A closer look at the Greaves citation shows that it is an opinion essay rather than an empirical study, so I don't think this statement is supported by the citation provided.	*We agree that social media is widely used in health care research today. Therefore, we decided to remove the sentence. We did find that research on (content as a part of) hashtag activism is relatively new, therefore we added a sentence (+reference) to the Introduction section, see page 5 line 113 to 115.*
Similarly, the authors argue that "social media research" is "a relatively new and innovative field." This statement is simply not true – the phenomenon of "social media" is at the center of vast quantities of research, including specifically in health care contexts. Furthermore, this research does not use any particularly innovative techniques (i.e. big data analysis). This does not mean that the research is bad! Quite the contrary, it appears to be good, useful research. My point is that the authors' argument that this is groundbreaking or innovative research detracts and is not necessary.	*We understand the comment of the reviewer. As stated above, we agree that social media is more and more used in health care research today. Therefore, we changed the statements we made around social media research and focused more on the use of hashtag activism content, see page 27 line 571 to 572.*
This following passage doesn't seem to make sense and should be cleaned up: "… they should not make us doubt that the #GG stories give good, and rare, first-person insight into how women were in fact affected and felt."	*We adjusted the sentence to:*<br><br>*''Whilst these considerations should make us wary of jumping to conclusions about causes or caregivers' knowledge, intention, or insight, the #GG stories provide us with useful insight into the negative and traumatic experiences from the women's point of view.''*<br><br>*see page 28 line 595 to 597.*
