# Supplementary material for: Left powerless: A qualitative social media content analysis of the Dutch #breakthesilence campaign on negative and traumatic experiences of labour and birth
Source: PLoS One. 2020 May 12;15(5):e0233114. doi: 10.1371/journal.pone.0233114 (PMC7217465; doi:10.1371/journal.pone.0233114)
Supplement: Attachment — Submitted filename: Response to Reviewers.pdf [file pone.0233114.s002.pdf]

24 April 2020

Manuscript Number: PONE-D-19-33121R1

Dear Mr Fischer,

We are happy to hear our manuscript entitled *Left powerless: a qualitative social media content analysis of the Dutch #breakthesilence campaign on negative and traumatic experiences of labour and birth* is almost suitable for publication.

We would like to thank reviewer #2 for his/her compliments on the adjustments we made to the Methods section and for the opportunity to respond to the final comment that was made:

The only aspect that gives me some pause is the passage: "In total 533 stories were collected, of which 95 were excluded." Why were these 95 excluded? Earlier you write that stories "shared by caregivers, partners or other witnesses of negative or traumatic experiences were excluded." Does that account for the 95? I might recommend adding just a few words about those exclusions to the passage quoted above, connecting it to that earlier explanation.

The following stories were excluded from the analysis: duplicate stories, stories only expressing gratitude to the movement and stories that were written from partners' or caregivers' perspectives. Behind the passage quoted by the reviewer, a reference to Figure 1 providing the reasons for exclusion of the 95 stories is given. As suggested by the reviewer, we added the following sentence for clarification: *The excluded stories consisted of duplicate stories (41), stories only expressing gratitude to the movement (12) and stories that were written from partners' (14) or caregivers' perspectives (28)*. (page 11, line 206-207).

We hope you are in agreement with this final edit and look forward to your reply regarding publication.

Yours sincerely, on behalf of the authors,

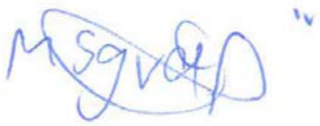

Marit van der Pijl, MSc

E: m.vanderpijl@amsterdamumc.nl (MP)

T: +31650892910
